# A high-throughput ResNet CNN approach for automated grapevine leaf hair quantification

**DOI:** 10.1038/s41598-025-85336-0

**Published:** 2025-01-10

**Authors:** Nagarjun Malagol, Tanuj Rao, Anna Werner, Reinhard Töpfer, Ludger Hausmann

**Affiliations:** Julius Kühn Institute (JKI), Federal Research Centre for Cultivated Plants, Institute for Grapevine Breeding Geilweilerhof, 76833 Siebeldingen, Germany

**Keywords:** Grapevine, Leaf hair, Phenotyping, CNN, *Vitis*, Plant sciences, Computational biology and bioinformatics, Machine learning

## Abstract

The hairiness of the leaves is an essential morphological feature within the genus *Vitis* that can serve as a physical barrier. A high leaf hair density present on the abaxial surface of the grapevine leaves influences their wettability by repelling forces, thus preventing pathogen attack such as downy mildew and anthracnose. Moreover, leaf hairs as a favorable habitat may considerably affect the abundance of biological control agents. The unavailability of accurate and efficient objective tools for quantifying leaf hair density makes the study intricate and challenging. Therefore, a validated high-throughput phenotyping tool was developed and established in order to detect and quantify leaf hair using images of single grapevine leaf discs and convolution neural networks (CNN). We trained modified ResNet CNNs with a minimalistic number of images to efficiently classify the area covered by leaf hairs. This approach achieved an overall model prediction accuracy of 95.41%. As final validation, 10,120 input images from a segregating F1 biparental population were used to evaluate the algorithm performance. ResNet CNN-based phenotypic results compared to ground truth data received by two experts revealed a strong correlation with *R* values of 0.98 and 0.92 and root-mean-square error values of 8.20% and 14.18%, indicating that the model performance is consistent with expert evaluations and outperforms the traditional manual rating. Additional validation between expert vs. non-expert on six varieties showed that non-experts contributed to over- and underestimation of the trait, with an absolute error of 0% to 30% and -5% to -60%, respectively. Furthermore, a panel of 16 novice evaluators produced significant bias on set of varieties. Our results provide clear evidence of the need for an objective and accurate tool to quantify leaf hairiness.

**Supplementary Information:**

The online version contains supplementary material available at 10.1038/s41598-025-85336-0.

## Introduction

In grapevine research, leaf hair has been used in ampelographic studies as one of the descriptors to identify and define various cultivars^1^. However, they have more biological features. Depending on the type and density of the hair on the leaves’ lower side, it can play a beneficial role concerning vine health^2^. Like other agricultural crops, grapevines (*Vitis vinifera *ssp.) are susceptible to several pests and fungal pathogens, causing extreme yield losses and quality reduction^3,4^. Some wild *Vitis *accessions prevent the attack utilizing physico-chemical factors and genetic resistances^5,6^. To date, several genetic loci have been identified conferring resistance to fungal pathogens^7–9^. *Plasmopara viticola *((Berk. and Curt.) Berl. and de Toni), the causative agent of downy mildew, infects all green parts of the plant, with the stomata on the lower leaf surface being the entry site for infection. Recent studies have shown the importance of grapevine abaxial leaf hair as a natural physical barrier against downy mildew infection^5,10^. Quantitative trait loci (QTL) for leaf hair density identified on four linkage groups of grapevine were also associated with resistance to downy mildew^11,12^. Furthermore, limited infection with anthracnose (*Elsinoë ampelina*) on the abaxial vs. adaxial leaf surface was observed due to high leaf hair density on the lower leaf surface that trapped conidia^13^. A major QTL in grapevine was identified, explaining the relevance of leaf hair density associated with the abundance of a predatory mite (*Typhlodromus pyri*) acting as a biological control agent^14^. Despite the high relevance, leaf hair density has so far only been rated visually^14,15^, and no previous studies have clearly reported or illustrated accurate and efficient means for measuring leaf hair density. Therefore, studies aiming to identify the correlation between leaf hair density and pests or pathogens tend to create biased data, due to high subjectivity in leaf hair phenotyping.

In recent years, advances in machine-learning-based image analysis have been extensively implemented in plant trait phenotyping and growth monitoring studies^16–19^. In particular, convolutional neural networks (CNNs) with varying numbers of layers and increased image classification depth have been successfully used in a wide range of biological classification systems^20–24^. In contrast to CNNs with lower numbers of layers like AlexNet, VGG-13, VGG-16, GoogLeNet, DenseNet169, and PReLU-net, the Residual Network (ResNet)-based CNN architecture has dense layers with fewer parameters, a minor error rate, and skip connections making it an intriguing choice for more difficult tasks^25^.

Empirical studies and benchmarks have consistently demonstrated that ResNet architectures outperform traditional CNN architectures in various computer vision tasks, including plant disease classification^26–29^. The architecture of ResNet enables the reuse of learned features across different layers. This reuse property enhances the network’s ability to generalize to unseen data, contributing to improved classification performance, particularly in scenarios with limited training data^25,30,31^. Currently, in grapevine research, several CNN-based tools are available demonstrating the power and efficiency of this technology in supporting qualitative and quantitative phenotyping of numerous traits^32–36^.

The main objective of this work was the development and implementation of an automated phenotyping tool based on ResNet CNN for the objective detection and quantification of grapevine leaf hairs. Therefore, a minimal set of training images and computational resources were used to evaluate the effectiveness of the CNN model in real-world applications. A leaf hair ResNet aims to enhance accuracy, reduce subjectivity, and increase throughput of leaf hair quantification. This ResNet CNN-based approach for leaf hair quantification enables better evaluation and helps to implement this morphological trait in grapevine breeding as a natural physical barrier.

## Materials and methods

### Plant material

A biparental F1 population of 496 progenies derived by the cross between ‘Morio Muskat’ (‘Silvaner Grün’ × ‘Muscat à Petits Grains Blancs’) and COxGT2 (*Vitis coignetiae* x ‘Gewürztraminer’), exhibiting quantitative segregation for the trait leaf hair density were utilized for image capturing. In addition, three non-hairy genotypes, ‘Riesling’, ’Regent’ and ‘Cabernet Sauvignon’ and three hairy genotypes, ‘Pinot Meunier’, ‘Tigvoasa’, and a *V. thunbergii* x *V. vinifera* hybrid, were also included in the image capturing process. The individuals of the cross population were grown in pots and maintained under greenhouse conditions at Julius Kühn-Institut (JKI), Siebeldingen, Germany. The six additional genotypes were grown under field conditions. The grapevine material used was taken from the own grapevine *ex situ* repository at JKI, Siebeldingen, in accordance with the relevant regulations and legislation (for material details see Supplementary Table 1; https://www.vivc.de).

### Experimental design

#### Leaf disc preparation and automated image acquisition

**Fig. 1:**
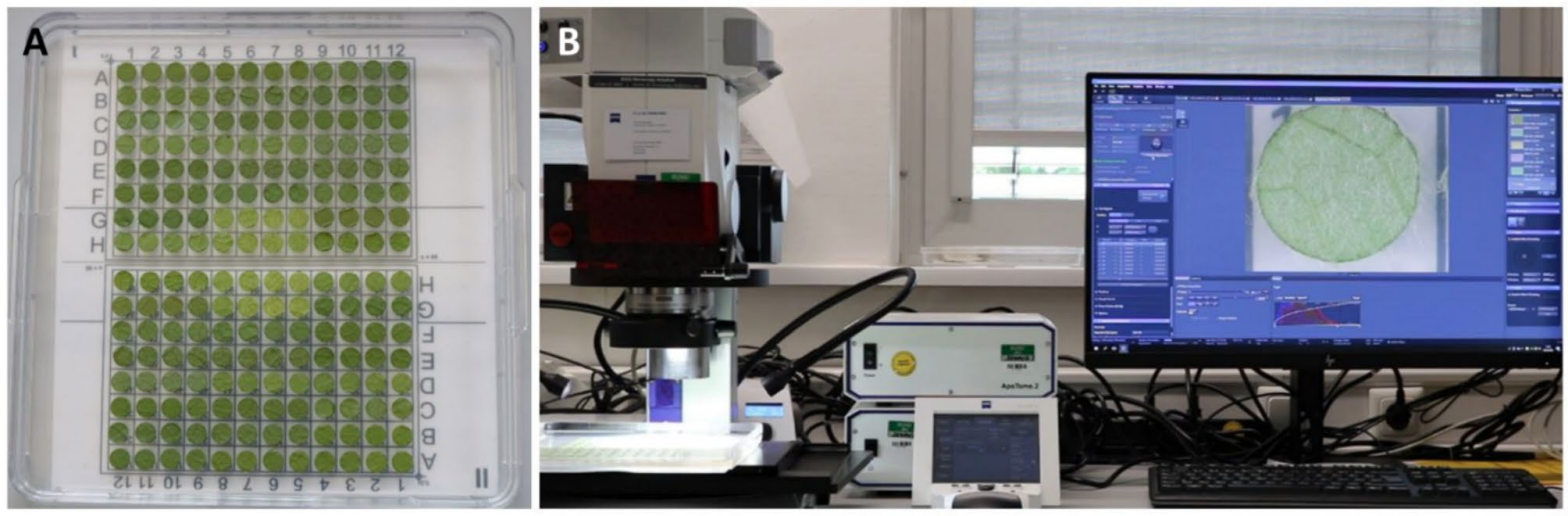
Leaf disc imaging. The layout template for leaf discs (**A**) combined with Zeiss^®^ Axio Zoom stereomicroscope (**B**) supported by a motorized table allows high-throughput image acquisition.

A single leaf was sampled from the third or fourth node below the shoot apex from each individual plant. Four leaf discs with 1 cm diameter were excised from each collected leaf using a cork borer. Leaf discs were placed on 1% water agar (Insula Agar Agar Pulver E406, Gustav Essig GmbH & Co. KG, Mannheim, Germany) in 245 mm Square BioAssay Dishes (Corning^®^, Corning, New York, NY, USA) according to a specific layout template, ensuring the leaf’s adaxial (upper surface) on the agar side (Fig. 1A).

Images of leaf discs were captured using the Zeiss^®^ Axio Zoom V16 stereomicroscope (Jena, Germany) coupled with a motorized table, enabling movement in the X and Y-axis (Fig. 1B). This fulfills the objective of high throughput with 96 discs/13min. Images were captured at 10.5 fold magnification with a 0.5x magnification lens (PlanApo Z 0.5×/0.125; Free Working Distance 114 mm), contributing to overall system magnification. For sample illumination, a combined light system consisting of a K-LED segmentable ring light, two gooseneck lights at a 45° angle, and a backlight provide a combined exposure time of 18–20 ms/image. JPG was used as the default image format with an image resolution of 6.08 mega pixels (size = 2752 × 2208) and a width/height ratio of 1.25. The ZenBlue version 3.4 (Zeiss, Jena, Germany) program was used to produce a movement template that enabled automated access to the 96 positions. In addition to the motorized table, the Zeiss Axio Zoom (Jena, Germany) V16 has a software autofocus that was utilized to determine the best focal height for each leaf disc prior to imaging. The lowest aperture feasible was used to get the most significant focal depth. For each leaf disc, a single picture was captured and uploaded to the Linux-based server for the model training. A complete detailed and installable workflow can be found with the protocol in GitHub (https://github.com/1708nagarjun/ResNet-CNN-Leaf-hair) (Zeiss Axiozoom users will be able to integrate the template easily in few steps).

### OIV-based manual evaluation of leaf hair density

**Fig. 2:**
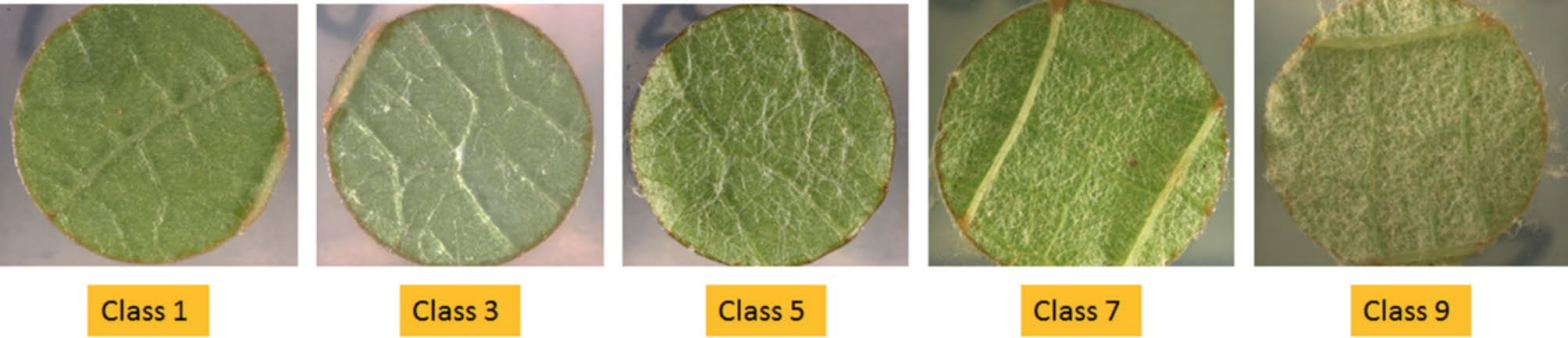
Classification scale for leaf hair density. Exemplary leaf discs of F1 individuals of ‘Morio Muskat’ x COxGT2 assigned to the five classes of the OIV descriptor 084.

The density of abaxial (lower surface) leaf hair present on the leaf discs was visually evaluated using the established OIV descriptor 084^37^. It was used to categorize the leaf hair density in five classes: Class 1: None/Very low; class 3: Low 5: Medium 7: High 9: Very high density of leaf hair. Each leaf disc image captured was assigned to the respective class (Fig. 2). All the leaf disc images captured from the F1 individuals of the cross ‘Morio Muskat’ x COxGT2 were classified according to the OIV descriptor 084.

### ResNet CNN model development

#### Data preprocessing

**Fig. 3:**
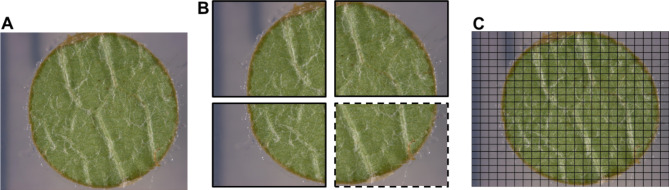
Image preparation for CNN. Start with images of one leaf disc (**A**). Each image was divided into four equal portions, ¾ parts used for training (with bold borders) and ¼ (dotted border) used for testing the model (**B**). As input image for the CNN model training served 506 individual image slices (**C**).

All the leaf disc images captured from the F1 individuals of the cross ‘Morio Muskat’ x COxGT2 were classified according to the OIV descriptor 084. Then a set of 20 leaf disc images, with four of them representing each of the five OIV 084 classes, was chosen to form the training and testing set for the CNN. Each leaf disc image (Fig. 3A) was sliced into 506 individual image slices with a resolution of 119 × 100 pixels (Fig. 3C) using a Python-based script (chop_images.py), out of which 75% were used as part of the training set, and the remaining 25% were used to form the testing set (Fig. 3B). To enhance the model’s robustness and prevent overfitting, data augmentation was performed by rotating the image slices and incorporating images captured under varying lighting conditions on different days and experiments. All the image slices were manually classified by two experts into the three classes: background (agar), leaf without hair, and leaf with hair using a Python script called image_sorter2_script.py (GitHub repository).

### Model building

**Fig. 4:**
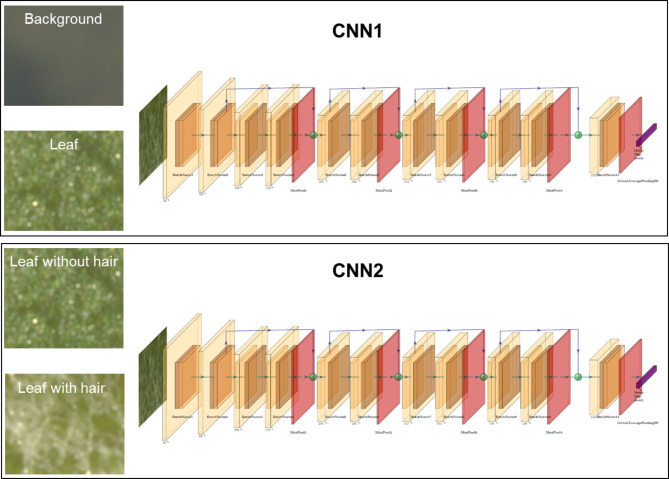
Detailed representation of the two ResNet CNNs architectures. CNN1: Background vs. leaf. CNN2: Leaf without hair vs. leaf with hair.

To improve accuracy, two sequential CNNs were developed instead of a single multi-class model. The first CNN classifies regions as “background” or “leaf disc,” while the second classifies the leaf disc as “with hair” or “without hair.” This stepwise approach simplifies the task, enhances accuracy, and reduces complexity. To avoid any subjective errors during manual labelling, the data for testing and validation were separated systematically, ensuring that the background vs. leaf classification and leaf with vs. without hair classifications were handled independently. This approach minimizes the risk of high error rates typically associated with multi-class models that attempt to classify all three categories simultaneously. A ResNet-based CNN architecture was chosen for both the CNNs used in this pipeline (Fig. 4). The first ResNet CNN has the classes “background” and “leaf disc”, from now on referred to as CNN1. It was trained with 10,089 individual image slices as part of the training set and 4,323 individual image slices as part of the validation set. The second ResNet CNN has the classes “leaf with no hair” and “leaf with hair,” from now on referred to as CNN2. It was trained with 4,369 image slices as part of the training set and 1,872 image slices as part of the validation set (Fig. 3B). All datasets and the code to train the CNNs are available in the GitHub repository. The CNNs were trained in Jupyter Notebook, on Google Colaboratory instances running Ubuntu 18.04.5 LTS, a 2-core Xeon CPU at 2.20 GHz, 13.3GB RAM and an Nvidia Tesla K80 GPU with 12GB memory. All CNN calculations were performed on the GPU.

The detailed summary of the CNN layers can be found on the GitHub repository (hair-nohair-model-summary.txt; Fig. 4). For CNN1, each slice in the size of (112 × 112) was loaded with RGB channels. A rescaling layer was used to scale the RGB values lying between 0 and 255 down to 0 and 1, followed by application of two blocks consisting of a 2D convolution layer, a batch normalization layer, followed by an activation layer using ReLU. The convolution layer size for block one was 32 and for block two was 64. After the first two blocks, the residual was set aside for future use. Four more blocks were added with separable 2D convolutions of sizes 128, 256, 512, and 728. Each block consists of an activation layer (ReLU), a separable 2D convolution layer, a batch normalization layer, another activation layer, another separable 2D convolution layer, another batch normalization layer, a 2D max pooling layer, and a final 2D convolution layer with the previous block residual as input. This result is then added back to the current values, which is again set aside for use in the next block. After the four blocks above, another block consisting of a separable 2D convolution layer of size 1024, a batch normalization layer, and an activation layer are added, the result of which is passed through a global 2D average pooling layer, followed by a dropout layer with rate 0.5. Finally, the regular deeply connected dense layer with 1 unit and sigmoid activation followed to get an output value between 0 and 1 where 0 is “background” class and 1 is “leaf disc” class (Fig. 4).

For CNN2, the same model architecture was used as for CNN1, but the dropout rate was changed to 0.2 (Fig. 4). For both the CNNs, the Adam optimizer with a learning rate of 0.001 and loss calculation was applied with the binary_crossentropy function. Both CNNs were trained for 30 epochs each.

### Optimizing parameters for the CNNs

Prototyping was used to determine the dropout rate, number of ResNet blocks, and optimizer settings. Multiple variations of the model were trained on the same dataset and compared to the results with the ground truth data derived by the two independent experts who classified each individual slice manually. During training, the models were checkpointed at each epoch, and the epochs with the smallest difference between training and validation accuracy were compared to the ground truth findings. The final image datasets and the Jupyter Notebook containing the code necessary for training of the final version of the CNNs are available on the GitHub repository.

### Performance evaluation

#### Ground truth: model validation by experts

To evaluate the performance accuracy of the model, 20 new leaf disc images (4 images per OIV 084 class) from F1 individuals of the cross ‘Morio Muskat’ x COxGT2 were analyzed using the ResNet-based CNN model. Simultaneously, the two experts categorized the individual image slices (10,120) into the three classes “background”, “leaf with hair” and “leaf without hair”. Additionally, they also visually rated the whole leaf disc images for percentage of leaf area covered with hair. Thus, two experts classified each leaf disc image at the slice level and manual rating (0–100%). The output results of the ResNet CNN (%) were correlated (Pearson correlation) with the ground truth data generated by manual sorting of slices (%), and manual rating of the whole leaf disc image (%) by expert 1 and expert 2, respectively.

#### Comparison between experts and non-experts

A set of three genotypes with non-hairy leaves (‘Riesling’, ’Regent’, ‘Cabernet Sauvignon’) and three genotypes with hairy leaves (‘Pinot Meunier’, ‘Tigvoasa’, and *V. thunbergii* x *V. vinifera*) were used for comparing the variability between two experts and two non-experts. The four evaluators each generated ground truth data by manually sorting individual image slices (3,036) and visually rating the whole leaf disc images (0–100% leaf hair density). Considering the ResNet CNN output (%) as true values and evaluator values as measured, absolute accuracy error (absolute error) was calculated^38^ for all the individual images.

#### Audience validation

Manual rating (0–100% leaf hair density) of the leaf disc images of the genotypes with non-hairy leaves (‘Riesling’, ’Regent’, ‘Cabernet Sauvignon’) and the three genotypes with hairy leaves (‘Pinot Meunier’, ‘Tigvoasa’, *V. thunbergii* x *V. vinifera)* was carried out by 16 inexperienced evaluators. The leaf disc images were displayed using a projector in a meeting room with all 16 evaluators being present at the same time. A detailed explanation of manual rating was provided by an expert displaying three trial leaf disc images, which varied in leaf hair density (0%, 55%, and 99%, respectively). Absolute accuracy errors were calculated for each individual image between the inexperienced evaluators, to compare and reveal high variability caused due to subjectivity.

## Results

### Model training and testing results

**Fig. 5:**
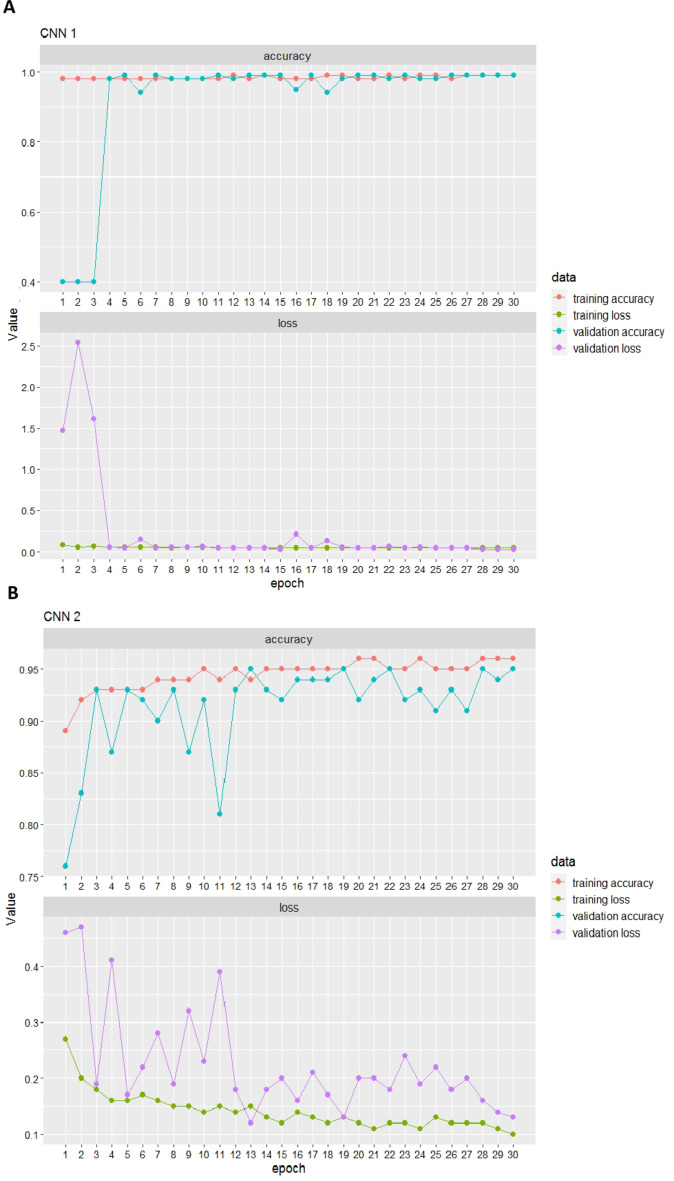
CNN accuracy. Training and validation accuracy, training and validation loss performances of CNN1 “background” vs. “leaf disc” (**A**) and CNN2 “leaf without hair” vs. “leaf with hair” (**B**) after 30 epochs.

To improve the phenotyping of the leaf hair density on the lower side of grapevine leaves, automated high-throughput imaging of leaf discs was first established. These images were then used to train and later validate a ResNet model, consisting of two CNNs. The first ResNet CNN “background” vs. “leaf disc” (CNN1) achieved an overall validation accuracy of 98% with a minor value of validation loss after 30 epochs (Fig. 5A). Whereas, the second ResNet CNN “leaf with no hair” vs. “leaf with hair” (CNN2) achieved an overall validation accuracy of 95% and validation loss of 13% after 30 epochs (Fig. 5B). CNN1 has a higher model validation accuracy compared to CNN2 due to the reduced feature complexity of the input slices. Therefore, the CNN2 model was more comprehensive in extracting the features resulting in slightly reduced validation accuracy. However, no overfitting or underfitting of the models was observed and both models achieved satisfying overall validation accuracy.

### Ground truth: experts validation

**Fig. 6:**
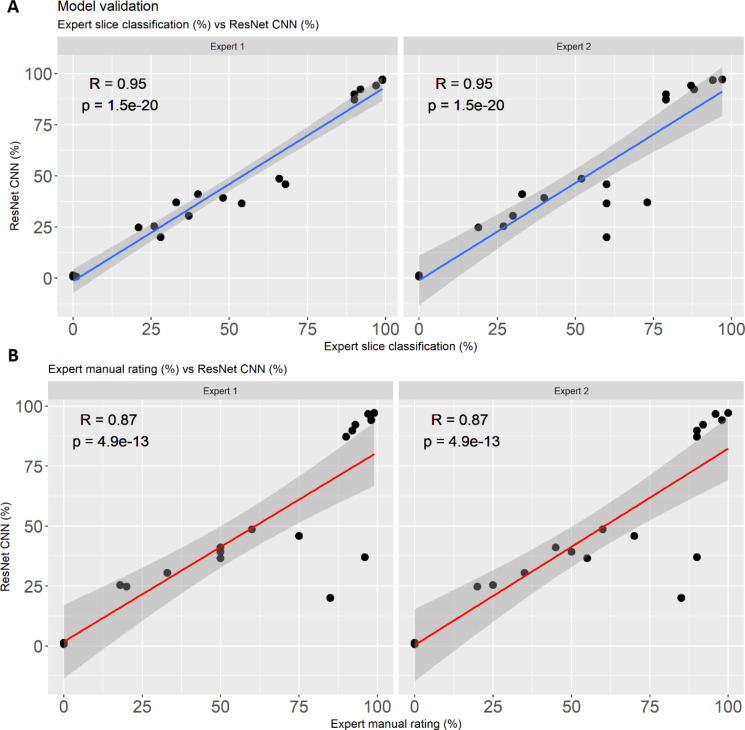
Validation 1 of experts vs. ResNet CNN. Correlation between two experts slice classification (%) vs. ResNet CNN evaluation (%) (**A**) and expert’s manual rating (%) vs. ResNet CNN evaluation (%) (**B**) of leaf disc images.

Once the training and testing of the ResNet CNNs was completed, the models were deployed to validate 20 (10,120 image slices) new leaf disc images (4 images per OIV 084 class) (Supplementary Fig. 1). These images were obtained from completely independent experiments conducted on separate days or months, ensuring that each image/image slice for each class originated from a distinct experimental setup. This approach strengthens the robustness and generalizability of the model. Again the two experts independently evaluated the identical set of images by classifying the slices and manually rated the leaf discs for the trait leaf hair (0–100%), hence generating the ground truth data. The independent ground truth datasets generated by the two experts are highly comparable to each other (Supplementary Table 2). The ResNet CNN output data (%) for the 20 images is in close agreement with the ground truth data generated by the two experts. In addition, the ResNet CNN output data (%) appeared to be extremely comparable to the slice sorting data in both experts’ evaluations, demonstrating the accuracy and sensitivity of the model. There are, however, a few inconsistencies such as image 1 in OIV 084 class 9 (Supplementary Fig. 1) being evaluated lower by the ResNet CNN than by expert 1 due to poor image features, uneven lighting, and image quality. The correlation between expert slice classification (%) for both experts and the ResNet CNN output data showed highly significant R values of 0.98 (*p* < 0.001) and 0.92 (*p* < 0.001), respectively, and Root Mean Square Error (RMSE) values of 8.20% and 14.18%, respectively (Fig. 6A). Whereas a slightly less significant correlation with R values of 0.86 (*p* < 0.001) and 0.87 (*p* < 0.001) and with RMSE of 21.43 and 20.37 were obtained between manual rating (%) of the experts and the ResNet CNN output data (%), respectively (Fig. 6B).

Overall, the correlation in the classification at slice level proved to be more precise than the manual rating correlation of both experts. There was a higher degree of subjectivity between the manual rating (%) in comparison to slice level classification (%) in the results of both experts, indicating the necessity for an objective classification model (Fig. 6). The correlation coefficient varied the most for leaf discs densely covered with leaf hair (70–90%), affecting the overall correlation. Therefore, it is crucial to use objective machine vision to precisely define quantitatively segregating features such as leaf hair. The correlation of ResNet CNN (%) and expert slice classification (%) for both experts (i.e., *R* = 0.98 and *R* = 0.92, respectively) clearly demonstrate the model’s accuracy and performance.

### Comparison between experts and non-experts

**Fig. 7:**
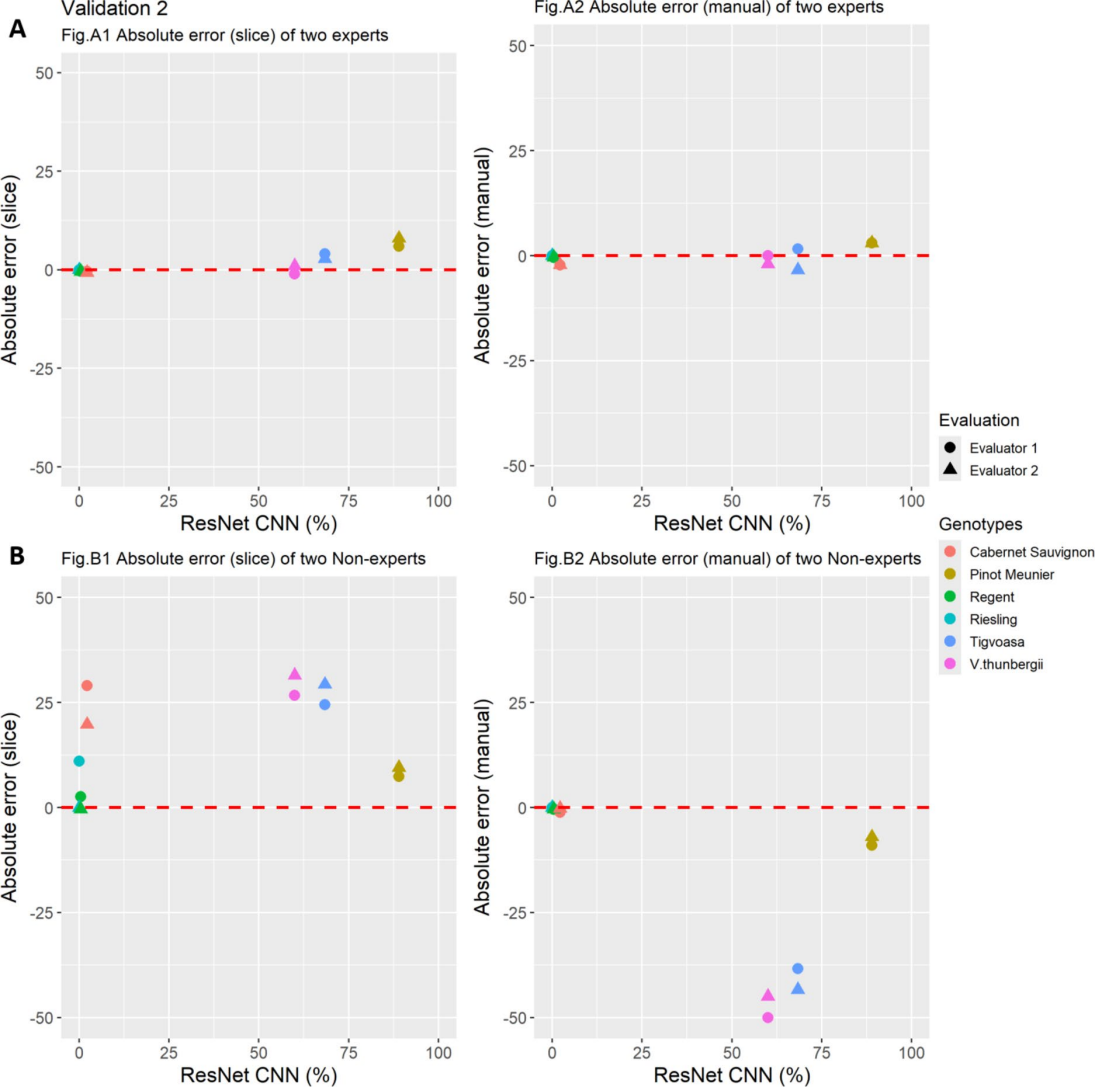
Validation 2 of experts vs. non-experts. Absolute error estimation of experts (**A)** and non-experts **(B)** for slice (%) and manual (%) rating, respectively. The red dotted (“0”) line represents the no error line. Filled circles represent evaluator 1 and filled triangles represent evaluator 2.

The ResNet CNN model was used to quantify the leaf hair of three genotypes with hairy leaves (‘Pinot Meunier’, ‘Tigvoasa’ and *V. thunbergii* x *V. vinifera*) and three genotypes with non-hairy leaves (‘Riesling’, ’Regent’ and ‘Cabernet Sauvignon’) (Supplementary Table 3), followed by absolute accuracy error (absolute error) estimation for the individual leaf disc images. The ResNet CNN output data (%) was considered as actual values and evaluator values, generated by a panel of two experts and two non-experts (Supplementary Table 4), as estimated. For the genotypes assessed, the absolute error variance was found to be highly negligible between the two experts for both the slice (%) and manual (%) ratings (Fig. 7A, A1 and A2).

The experts demonstrated greater precision and repeatability in the assessments performed. In the case of both non-experts, the absolute error variance ranged between 15% and 30% for slice classification (%), indicating a strong tendency to overestimate. In the manual rating (%), however, both the non-experts underestimated the leaf hair quantification, with the absolute error variance ranging between -20% to -50% (Fig. 7B, B1). Surprisingly, the three genotypes with hairy leaves were underestimated, showing that manual ratings by non-experts might introduce a significant bias (Fig. 7B, B2). These findings clearly stipulate the need for a technique for quantifying leaf hair that is objective, accurate, and precise.

### Audience validation

**Fig. 8:**
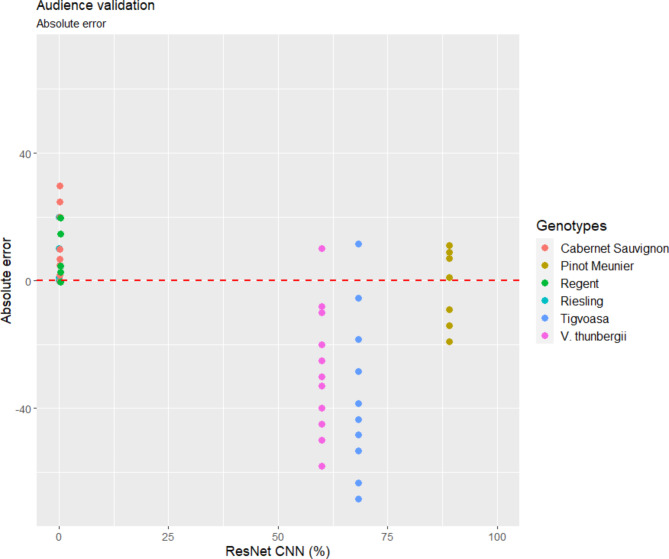
Validation 3 by audience. Absolute errors (Absolute Error = Measured Value - Actual Value) estimation of manual rating for a panel of 16 novice evaluators. Each dot represents the result of a single evaluation; the red dotted (“0”) line represents the no error line.

In the absence of a ResNet CNN classifier, untrained/novice evaluators tend to overestimate the non-hairy genotypes and underestimate the hairy genotypes (Fig. 8, Supplement Table 5). The overall significant bias in terms of absolute error was found to be between 0 and 30% for genotypes with non-hairy leaves, and 5 to 60% for genotypes with hairy leaves. Furthermore, it was obvious that the intermediate genotypes, *V. thunbergii* x *V. vinifera* and ‘Tigvoasa’, were challenging to rate (Supplement Table 6).

### Leaf disc classification pipeline and command line tool

The whole process of automated leaf hair evaluation was organized into a single command line tool that runs on a UNIX environment. The tool generates an output in two formats, one in the form of a csv files, which aggregate all the image results in the test, the other in the form of jpg images showing slice maps of the original input images for better visualization of the area covered with leaf hairs. The whole pipeline is publicly available on the open source repository GitHub (https://github.com/1708nagarjun/ResNet-CNN-Leaf-hair*).*

**Fig. 9:**
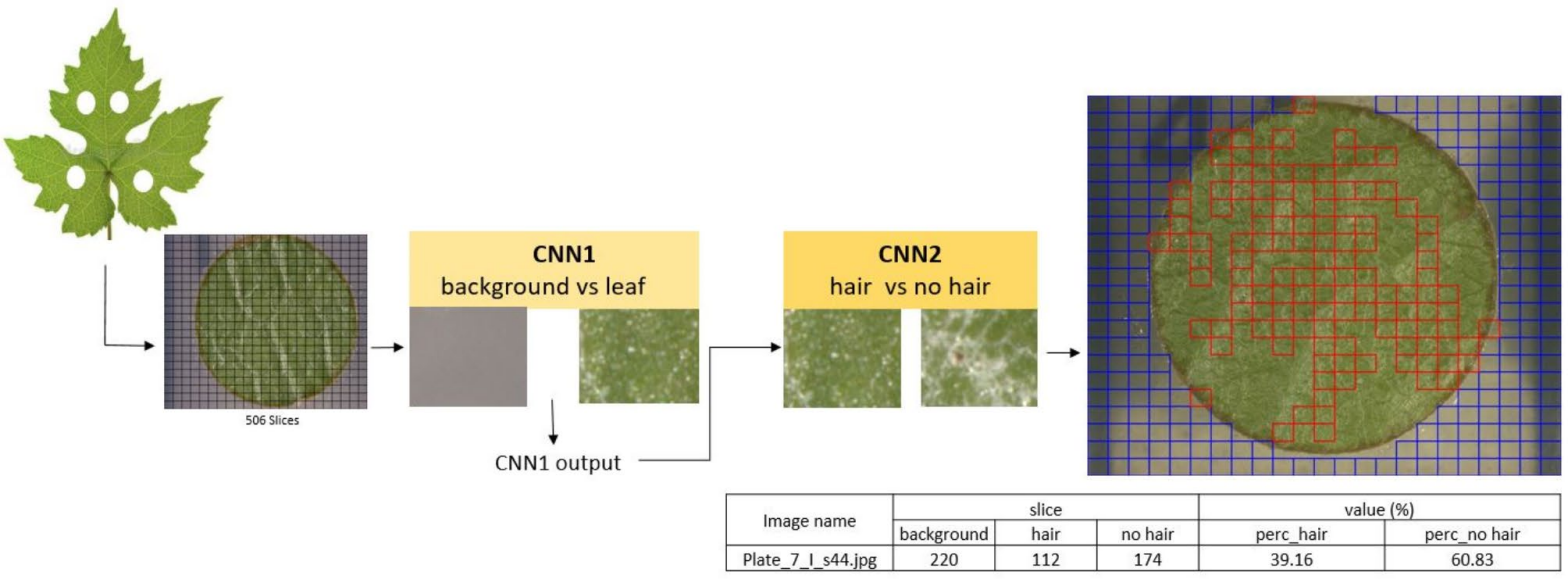
Outline of the ResNet-based leaf hair quantification pipeline. The final output is a table with percentage values and a slice map (.jpg), where the individual slices with leaf hairs are annotated in red. (img name: image name; back: slices identified as background/agar; hair: slices identified as hair contained; nohair: slices identified as no hair contained; perc_hair: area of leaf disc covered with hair (%); perc_nohair: area of leaf disc not covered with hair (%)).

First, four leaf discs are punched out of a leaf, imaged under a microscope and used as input data (refer methods section for details). The images are sliced into 506 slices, which are then classified by CNN1 into the two classes “background” or “leaf disc”. The leaf disc slices are further classified by CNN2 into the classes “leaf disc without hair” and “leaf disc with hair”. The final percentage of hair on the leaf disc is calculated by taking the proportion of slices classified as “leaf disc with hair” relative to the total number of slices classified as “leaf disc” by CNN2, ensuring a robust and accurate quantification of hair density. For each leaf image, the Python-based code (classifier.py) systematically creates 506 slices and saves their coordinates relative to the original image. After classification, the code rearranges the slices into their original order to generate a slice map (Fig. 9), which visually represents the classifications across the entire leaf disc. This process preserves spatial context, ensuring that no data about the distribution of hair density is lost. The slice map also enables visual verification of localized variations, providing a clear overview of the results while maintaining accuracy and scalability. The pipeline runs in a loop over every single image present in the input directory. After classification, the tool generates slice maps that overlay the input images with red boxes outlining the slices containing leaf disc area covered with hairs and blue boxes outlining the slices that contain the background. Finally, the results are summarized and saved in a csv file that contains the name of the image, number of slices with background, number of slices with hairs, number of slices with no hairs, percentage of leaf disc covered with hairs, and percentage of leaf disc not covered by hairs. The generated files, slice of the slice maps and result tables are time-stamped and stored in the output directory (Fig. 9). Detailed instructions about the leaf hair quantification tool are provided in the GitHub repository.

## Discussion

Quantifying plant functional traits using advanced imaging technology offers breeders a more efficient and progressive approach to assist their decision-making. To uncover the hidden genetics of quantitative traits, the availability of accurate and objective measurement methods is crucial in research. A recent trend in machine learning, such as CNNs, has been widely utilized in plant disease identification^39–42^. CNNs combined with digital images are a powerful and cost-effective method for monitoring and maximizing agricultural products^43,44^. Image analysis has already been demonstrated to be more accurate than visual estimation in a number of pathosystems^45–48^. CNNs have also been successfully implemented in the efficient quantification of grapevine diseases such as powdery mildew and downy mildew, which significantly improved the trait analysis^11,32,34^. Numerous convolutional neural network (CNN) architectures, including VGG (Visual Geometry Group), Inception (GoogLeNet), DenseNet (Densely Connected Convolutional Networks), MobileNet, and ResNet (Residual Networks), have been meticulously developed and persistently advocated for their application in plant disease identification tasks^49–51^. In general, ResNet stands as an excellent choice for plant disease recognition due to its ability to efficiently capture intricate patterns in plant images while mitigating the degradation problem often encountered in deeper networks, thus ensuring high classification accuracy and robust performance in real-world applications. Its state-of-the-art performance, evidenced by numerous empirical studies, underscores ResNet’s superiority in handling the complexities inherent in plant disease classification tasks^52–54^. In this study, modified ResNet CNN was trained to quantify leaf hairiness on grapevine leaf discs using a minimalistic set of RGB images and computational resources. ResNet’s robust residual connections enable accurate feature extraction and generalization, making it well-suited for this task, particularly in laboratory-based studies requiring precise classification. The ResNet CNN tool achieved an overall model accuracy of 95% and validation loss of 13%. It was used in a real-world application and demonstrated its ability to recognize and quantify leaf hair. The evaluation performance of the proposed ResNet CNN model in comparison with the ground truth data generated by two experts showed a significant correlation of *R* = 0.98 and *R* = 0.92, respectively (Fig. 6). Furthermore, the comparison of the manual scoring of the experts according to the OIV descriptor 084 with the ResNet CNN results also showed a high level of agreement (Fig. 6). This implies the ability of the ResNet CNN to learn complex features from RGB images and to transfer it with slightly negligible decreased accuracy. In addition, the validation with a complex practical case, images of leaf discs from six varieties with different genetic backgrounds, showed the high performance of the ResNet CNN model. The true values of the ResNet CNN model showed only a minimal deviation from the assessments of the experts (Fig. 7A). In contrast, significant deviations from true values were detected in the non-expert data, resulting in over- and underestimations and affecting the overall phenotyping accuracy (Fig. 7B). This observation was also confirmed by an audience-based validation performed to determine the bias novice evaluators can cause. Varieties with hairless leaves were clearly overestimated, varieties with hairy leaves underestimated, and considerable inconsistent deviations were found between the evaluators (Fig. 8). The data provided clearly suggest that for a quantitative trait like leaf hair, the competence of a trained expert is a prerequisite to get objective phenotypic data. Because the evaluations of non-experts and beginners contribute to a high variance, subsequent analysis such as QTL or GWAS (genome-wide association study) are also strongly affected. This indicates the necessity to implement machine-based vision for objective quantification of leaf hair. In comparison to established classical approaches, the developed ResNet CNN model has outperformed the novice and competent evaluators in leaf hair quantification for different cultivars. While this study focused on developing a robust and accurate tool for quantifying leaf hair density, we recognize the potential biological implications of this trait in processes such as disease resistance, pest deterrence, or other agronomic traits. This model provides a robust foundation for researchers aiming to explore and establish connections between leaf hair density and related traits.

### Limitation of work and future perspective

The trained ResNet CNN demonstrated high accuracy and efficiency; however, the first CNN model (CNN1) is specifically designed for an agar background. While it is true that some laboratories might use tinted or colored agar, this is a rare exception. The majority of studies, including ours, use neutral-colored agar, which is the standard in laboratory-based assays. This ensures compatibility with our model and aligns with commonly accepted practices. Any variation in background color or material other than agar may affect the overall accuracy and lead to discrepancies in quantification. In the presented approach, the leaf hairs are quantified on leaf discs and not on whole leaves, but the results are on average consistent with those of whole leaves. Small leaf discs are advantageous, since leaf hairs are small in structure and capturing images of the whole leaf is more difficult. Lower resolution or lower quality images could affect model performance and contribute to bias. Nevertheless, leaf hair on grapevine leaves are uniformly distributed; three to four leaf discs from a single leaf should correspond to the overall percentage of hair coverage. This study offers a simple and efficient approach to quantifying leaf hair density using ResNet-based CNN analysis of leaf disc images. Unlike segmentation methods, which may introduce bias or require microscopic images for densely haired varieties, this model provides a robust and objective solution for reliable phenotyping. Yet, categorical classification approach can be the future work. In addition, segmentation or semantic labelling of different types of leaf hair examined on the leaf disc is a surpassing method of categorical classification.

## Conclusion

Accurate and precise phenotyping of quantitative traits such as leaf hair is challenging and laborious. This study provides an automated, valuable tool that will pave the way for an efficient, accurate and reliable quantification of leaf hair. The potential of the ResNet CNN was shown in real-world applications, reducing bias and increasing the reliability of the quantification compared to the results of untrained evaluators. It is highly suggested for quantification of leaf hair in the *Vitis* genus, including disease resistance, fungicide administration, and insect diversity studies. Thus, the ResNet CNN model is reliable, promising and significantly more advantageous because of its objectified machine vision for evaluating grapevine leaf hair. This tool can be utilized in screening of germplasm repositories and serves as a promising application in generating phenotyping data utilized in QTL and GWAS studies. We are convinced that the ResNet CNN model presented for grapevine leaves can be transferred to other crops as well as other parts of plants after adaption enabling the effective exploration of leaf hair dynamics across different plant species. CNN-based methods in general will become an indispensable tool for plant scientists around the world in the future, clearly improving and accelerating the way we phenotype plant traits.

## Electronic supplementary material

Below is the link to the electronic supplementary material.

Supplementary Material 1

## Data Availability

The script of the ResNet CNN along with the images are available in the GitHub repository: https://github.com/1708nagarjun/ResNet-CNN-Leaf-hair.

## References

[1] Gago, P. et al. Microanatomy of leaf trichomes: opportunities for improved ampelographic discrimination of grapevine (*Vitis vinifera* L.) cultivars. *Aust J. Grape Wine Res.* **22**, 494–503 (2016).

[2] Schmidt, R. A. Leaf structures affect predatory mites (*Acari*: Phytoseiidae) and biological control: a review. *Exp. Appl. Acarol*. **62**, 1–17 (2014). 23990040 10.1007/s10493-013-9730-6

[3] Leroy, P. et al. A bioeconomic model of downy mildew damage on grapevine for evaluation of control strategies. *Crop Prot.* **53**, 58–71 (2013).

[4] Reineke, A. & Thiéry, D. Grapevine insect pests and their natural enemies in the age of global warming. *J. Pest Sci.* **89**, 313–328 (2016).

[5] Kortekamp, A. & Zyprian, E. Leaf hairs as a basic protective barrier against downy mildew of grape. *J. Phytopathol.* **147**, 453–459 (1999).

[6] Toffolatti, S. L. et al. Phenotypic and histochemical traits of the interaction between *Plasmopara viticola* and resistant or susceptible grapevine varieties. *BMC Plant. Biol.* **12**, 1–16 (2012). 22852828 10.1186/1471-2229-12-124 PMC3509031

[7] Dry, I. et al. Scion breeding for resistance to biotic stresses. In **The Grape Genome** (eds Cantu, D. & Walker, M. A.) 319–347 (Springer, 2019).

[8] Vezzulli, S. et al. Genomic designing for biotic stress resistant grapevine. In *Genomic Designing for Biotic Stress Resistant Fruit Crops* (ed Kole, C.) 87–255 (Springer, 2022).

[9] Possamai, T. & Wiedemann-Merdinoglu, S. Phenotyping for QTL identification: a case study of resistance to *Plasmopara viticola* and *Erysiphe necator* in grapevine. *Front. Plant. Sci.* **13**, 930954 (2022). 36035702 10.3389/fpls.2022.930954 PMC9403010

[10] Kono, A. & Shimizu, T. Leaf trichomes as an effective structure for disease resistance: the case of grapevine downy mildew. *Jpn. Agric. Res. Q.* **54**, 293–298 (2020).

[11] Divilov, K. et al. Computer vision for high-throughput quantitative phenotyping: a case study of grapevine downy mildew sporulation and leaf trichomes. *Phytopathology* **107**, 1549–1555 (2017). 28745103 10.1094/PHYTO-04-17-0137-R

[12] Kono, A. et al. Development of SSR markers linked to QTL reducing leaf hair density and grapevine downy mildew resistance in *Vitis vinifera*. *Mol. Breed.* **38**, 1–19 (2018).

[13] Braga, Z. V. et al. Histopathology of infection and colonisation of *Elsinoë ampelina* on grapevine leaves. *Eur. J. Plant. Pathol.* **154**, 1009–1019 (2019).

[14] Barba, P. et al. A QTL associated with leaf trichome traits has a major influence on the abundance of the predatory mite (*Typhlodromus pyri*) in a hybrid grapevine population. *Hortic. Res.* **6**, 87 (2019). 31645947 10.1038/s41438-019-0169-8 PMC6804712

[15] Yin, L. et al. Fine mapping of leaf trichome density revealed a 747-kb region on chromosome 1 in cold-hardy hybrid wine grape populations. *Front. Plant. Sci.* **12**, 587640 (2021). 33746993 10.3389/fpls.2021.587640 PMC7965957

[16] Omari, M. K. et al. Digital image-based plant phenotyping: a review. *Korean J. Agric. Sci.* **47**, 119–130 (2020).

[17] Lu, J., Tan, L. & Jiang, H. Review on convolutional neural network (CNN) applied to plant leaf disease classification. *Agriculture* **11**, 707 (2021).

[18] Triki, A. et al. Deep leaf: mask R-CNN based leaf detection and segmentation from digitized herbarium specimen images. *Pattern Recogn. Lett.* **150**, 76–83 (2021).

[19] Bera, A., Bhattacharjee, D. & Krejcar, O. PND-Net: plant nutrition deficiency and disease classification using graph convolutional network. *Sci. Rep.* **14**, 15537 (2024). 38969738 10.1038/s41598-024-66543-7 PMC11226607

[20] Mohanty, S. P., Hughes, D. P. & Salathé, M. Using deep learning for image-based plant disease detection. *Front. Plant. Sci.* **7**, 1419 (2016). 27713752 10.3389/fpls.2016.01419 PMC5032846

[21] Rawat, W. & Wang, Z. Deep convolutional neural networks for image classification: a comprehensive review. *Neural Comput.* **29**, 2352–2449 (2017). 28599112 10.1162/NECO_a_00990

[22] Lei, F. et al. Shallow convolutional neural network for image classification. *SN Appl. Sci.* **2**, 1–8 (2020).

[23] Gutiérrez, S. et al. Deep learning for the differentiation of downy mildew and spider mite in grapevine under field conditions. *Comput. Electron. Agric.* **182**, 105991 (2021).

[24] Ahmed, S. F. et al. Deep learning modelling techniques: current progress, applications, advantages, and challenges. *Artif. Intell. Rev.* **56**, 13521–13617 (2023).

[25] He, K., Zhang, X., Ren, S. & Sun, J. Deep residual learning for image recognition. In: *Proceedings of the IEEE Conference on Computer Vision and Pattern Recognition (CVPR)*, IEEE, 770–778 (2016).

[26] Brahimi, M. et al. Deep learning for plant diseases: detection and saliency map visualisation. In *Human and Machine Learning* (eds Zhou, J. & Chen, F.) 93–117 (Springer, 2018).

[27] Zhang, Y. et al. ResViT-Rice: a deep learning model combining residual module and transformer encoder for accurate detection of rice diseases. *Agriculture* **13**, 1264 (2023).

[28] Kumar, V., Arora, H. & Sisodia, J. ResNet-based approach for detection and classification of plant leaf diseases. In: *Proceedings of the International Conference on Electronics and Sustainable Communication Systems (ICESC 2020)*, IEEE, 495–502 (2020).

[29] Kumar, S. et al. Performance evaluation of ResNet model for classification of tomato plant disease. *Epidemiol. Methods*. **12**, 20210044 (2023).

[30] Yang, X. et al. Learning to extract semantic structure from documents using multimodal fully convolutional neural networks. In *Proceedings of the IEEE Conference on Computer Vision and Pattern Recognition*, 5315–5324 (2017).

[31] Muslih & Krismawan, A. D. Tomato Leaf diseases classification using Convolutional neural networks with transfer learning Resnet-50. *Kinetik: Game Technol. Inf. Syst. Comput. Netw. Comput. Electron. Control* **9**, 149–158 (2024).

[32] Bierman, A. et al. A high-throughput phenotyping system using machine vision to quantify severity of grapevine powdery mildew. *Plant Phenomics* 2019, 9209727 (2019). 10.34133/2019/9209727 PMC7706338 33313539

[33] Liu, B. et al. Grape leaf disease identification using improved deep convolutional neural networks. *Front. Plant. Sci.* **11**, 1082 (2020). 32760419 10.3389/fpls.2020.01082 PMC7373759

[34] Zendler, D. et al. High-throughput phenotyping of leaf discs infected with grapevine downy mildew using shallow convolutional neural networks. *Agronomy* **11**, 1768 (2021).

[35] Fuentes, S., Tongson, E. & Gonzalez Viejo, C. New developments and opportunities for AI in viticulture, pomology, and soft-fruit research: a mini-review and invitation to contribute articles. *Front. Hortic.* **2**, 1282615 (2023).

[36] Karim, M. J. et al. Enhancing agriculture through real-time grape leaf disease classification via an edge device with a lightweight CNN architecture and Grad-CAM. *Sci. Rep.* **14**, 16022 (2024). 38992069 10.1038/s41598-024-66989-9 PMC11239930

[37] OIV. *OIV Descriptor list for Grape Varieties and Vitis Species* 2nd edn (Organisation Internationale de la Vigne et du Vin, Organisation Intergouvernementale, 2009).

[38] R Core Team. *R: A language and environment for statistical computing*. R Foundation for Statistical Computing, Vienna, Austria. (2021). https://www.R-project.org/

[39] Kamilaris, A. & Prenafeta-Boldú, F. X. A review of the use of convolutional neural networks in agriculture. *J. Agric. Sci.* **156**, 312–322 (2018).

[40] Li, Y., Nie, J. & Chao, X. Do we really need deep CNN for plant diseases identification? *Comput. Electron. Agric.* **178**, 105803 (2020).

[41] Boulent, J., Foucher, S., Théau, J. & St-Charles, P. L. Convolutional neural networks for the automatic identification of plant diseases. *Front. Plant. Sci.* **10**, 941 (2019). 31396250 10.3389/fpls.2019.00941 PMC6664047

[42] Thiagarajan, J. D. et al. Analysis of banana plant health using machine learning techniques. *Sci. Rep.* **14**, 15041 (2024). 38951552 10.1038/s41598-024-63930-y PMC11217365

[43] Jiang, Y. & Li, C. Convolutional neural networks for image-based high-throughput plant phenotyping: a review. *Plant Phenomics* 2020, 4152816 (2020). 10.34133/2020/4152816 PMC7706326 33313554

[44] Demilie, W. B. Plant disease detection and classification techniques: a comparative study of the performances. *J. Big Data*. **11**, 5 (2024).

[45] Barbedo, J. G. A. & Gracia, M. Image analysis for agricultural applications: a comprehensive review. *Crop Prot.* **50**, 32–40 (2013).

[46] Yang, B. & Xu, Y. Applications of deep-learning approaches in horticultural research: a review. *Hortic. Res.* **8**, 123 (2021). 34059657 10.1038/s41438-021-00560-9 PMC8167084

[47] Keras *The Python deep learning API.* URL: https://keras.io (2022). Accessed June 2022.

[48] Nagi, R. & Tripathy, S. S. Deep convolutional neural network based disease identification in grapevine leaf images. *Multimed Tools Appl.* **81**, 24995–25006 (2022).

[49] Aishwarya, M. P. & Reddy, P. Ensemble of CNN models for classification of groundnut plant leaf disease detection. *Smart Agric. Technol.* **6**, 100362 (2023).

[50] Kaya, Y. & Gürsoy, E. A novel multi-head CNN design to identify plant diseases using the fusion of RGB images. *Ecol. Inf.* **75**, 101998 (2023).

[51] Deb, M. et al. A CNN-based model to count the leaves of rosette plants (LC-Net). *Sci. Rep.* **14**, 1496 (2024). 38233479 10.1038/s41598-024-51983-y PMC10794187

[52] Ding, R. et al. Improved ResNet based apple leaf diseases identification. *IFAC PapersOnLine*. **55**, 78–82 (2022).

[53] Dawod, R. G. & Dobre, C. ResNet interpretation methods applied to the classification of foliar diseases in sunflower. *J. Agric. Food Res.* **9**, 100323 (2022).

[54] Li, W., Zhu, D. & Wang, Q. A single view leaf reconstruction method based on the fusion of ResNet and differentiable render in plant growth digital twin system. *Comput. Electron. Agric.* **193**, 106712 (2022).

