# Supplementary material for: A high-throughput ResNet CNN approach for automated grapevine leaf hair quantification
Source: Sci Rep. 2025 Jan 10;15:1590. doi: 10.1038/s41598-025-85336-0 (PMC11724064; doi:10.1038/s41598-025-85336-0)
Supplement: Supplementary file 1 — Supplementary Material 1 [file 41598_2025_85336_MOESM1_ESM.pdf]

Supplementary Information

Theses supplementary materials contain 6 Tables and 1 Figure.

A high-throughput ResNet CNN approach for automated grapevine leaf hair quantification

Nagarjun Malagol, Tanuj Rao, Anna Werner, Reinhard Töpfer and Ludger Hausmann*

Julius Kühn Institute (JKI), Federal Research Centre for Cultivated Plants,
Institute for Grapevine Breeding Geilweilerhof, 76833 Siebeldingen, Germany

Journal: Scientific Reports
DOI: 10.1038/s41598-025-85336-0

* Corresponding author: ludger.hausmann@julius-kuehn.de;

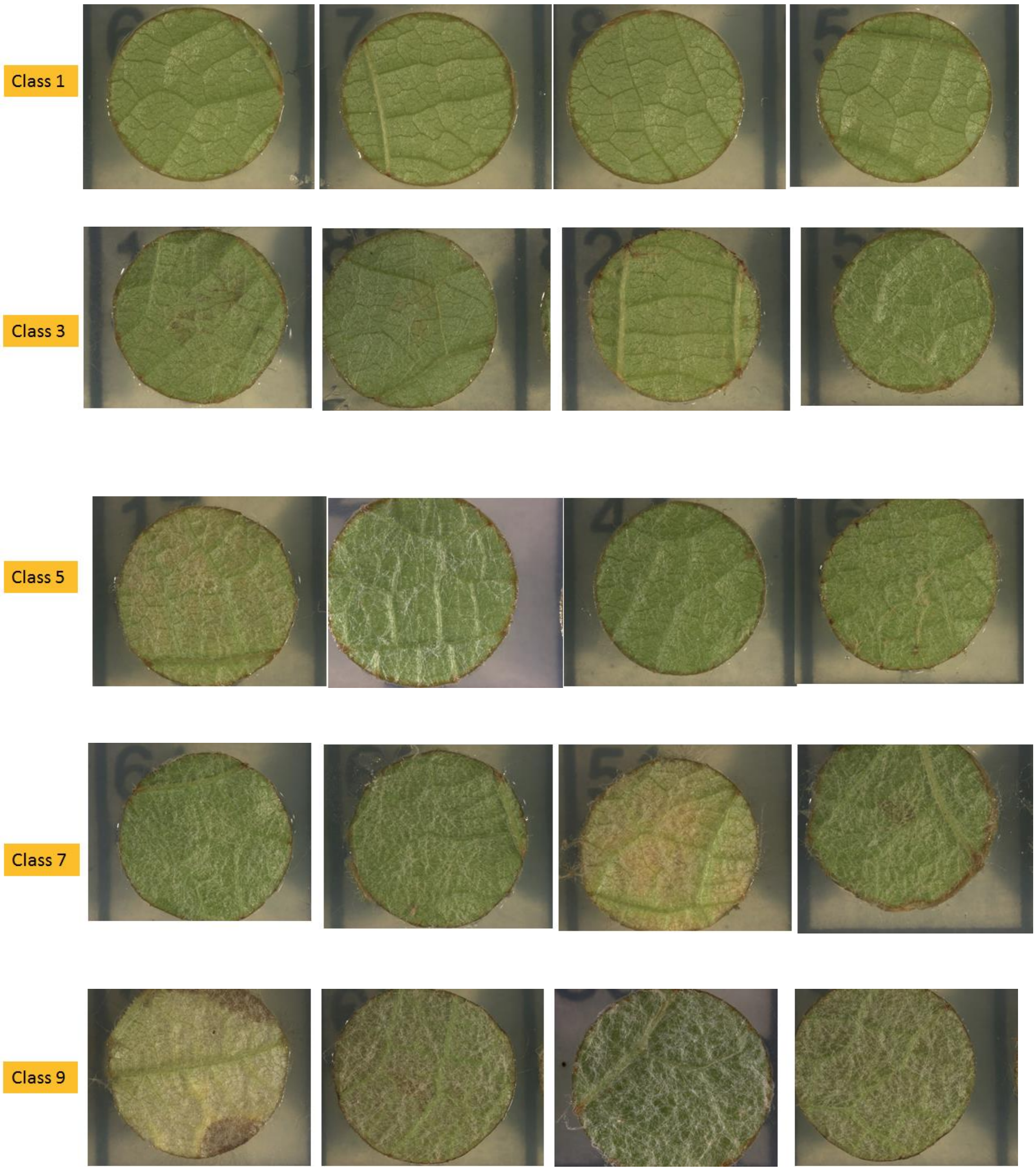

Supplementary Figure 1: Validation 1 (correlation of two experts and ResNet CNN): Leaf disc images used for the validation of the ResNet CNN model (four images for the five classes of the OIV leaf hair descriptor 086.

Supplementary Table 1. Grapevine accessions used, their metadata and their origin. *V*IVC=*Vitis* International Variety Catalogue (https://www.vivc.de); *V*=*Vitis*;

Prime name	Variety number *V*IVC	Accession name	Accession number	Origin	Remarks
Morio Muskat	7996	Morio Muskat	DEU098-1980-037	JKI Siebeldingen	
COxGT2	27137	COXGT2	DEU098-2021-027	JKI Siebeldingen	*V. coignetiae* x Gewürztraminer hybrid
Cabernet Sauvignon	1929	Cabernet Sauvignon	DEU098-1990-015	JKI Siebeldingen	
Pinot Meunier	9278	Pinot Meunier	DEU098-1980-041	JKI Siebeldingen	
Regent	4572	Regent	DEU098-1980-701	JKI Siebeldingen	
Riesling Weiss	10077	Riesling Weiss Cl. 90	DEU098-1980-316	JKI Siebeldingen	
Tigvoasa	17114	Veltliner Braun	DEU098-1980-370	JKI Siebeldingen	
V. thunbergii x *V. vinifera*	NA	*V. ficifolia* Cl. GF1	DEU098-1980-774	JKI Siebeldingen	Putativ *V. thunbergii* x *V. vinifera* hybrid

Supplementary Table 2: Validation 1: Single results of ResNet CNN (%) and experts evaluation of leaf discs; four images were analyzed for each of the five classes of OIV descriptor 086. Slice %: Slice classification ("background"/"Leaf with hair"/"Leaf without hair"). Manual %: Manual rating of leaf discs (0-100% leaf hair density)

		**Leaf disc #1**	**Leaf disc #2**	**Leaf disc #3**	**Leaf disc #4**
**Class 1**	ResNet CNN (%)	**1**	**0.7**	**1.4**	**0.7**
	Expert 1 (Slice %)	0	0	0	1
	Expert 2 (Slice %)	0	0	0	0
	Expert 1 (Manual %)	0	0	0	0
	Expert 2 (Manual %)	1	0	0	0
**Class 3**	ResNet CNN (%)	**25.4**	**24.7**	**30.5**	**48.6**
	Expert 1 (Slice %)	26	21	37	66
	Expert 2 (Slice %)	18	20	33	60
	Expert 1 (Manual %)	27	19	30	52
	Expert 2 (Manual %)	25	20	35	60
**Class 5**	ResNet CNN (%)	**45.8**	**41**	**39.2**	**36.5**
	Expert 1 (Slice %)	68	40	48	54
	Expert 2 (Slice %)	75	50	50	50
	Expert 1 (Manual %)	60	33	40	60
	Expert 2 (Manual %)	70	45	50	55
**Class 7**	ResNet CNN (%)	**87.2**	**89.8**	**20**	**92.2**
	Expert 1 (Slice %)	90	90	28	92
	Expert 2 (Slice %)	90	92	85	93
	Expert 1 (Manual %)	79	79	60	88
	Expert 2 (Manual %)	90	90	85	92
**Class 9**	ResNet CNN (%)	**37**	**94.1**	**96.7**	**97.1**
	Expert 1 (Slice %)	33	97	99	99
	Expert 2 (Slice %)	96	98	97	99
	Expert 1 (Manual %)	73	87	94	97
	Expert 2 (Manual %)	90	98	96	100

Supplementary Table 3: Validation 2: Summary of ResNet CNN (%) vs. panel of evaluations of six genotype leaf discs from two experts and non-experts. Slice %: Slice classification ("background"/”Leaf with hair”/”Leaf without hair”). Manual %: Manual rating of leaf discs (0-100% leaf hair density)

		Genotypes with hairy leaves			Genotypes with non-hairy leaves		
		V. thunbergii* x *V. vinifera	**Tigvoasa**	**Pinot Meunier**	**Riesling**	**Regent**	**Cabernet Sauvignon**
	ResNet CNN (%)	60.0	68,4	89	0	0.4	2.2
Evaluator 1	Expert 1 (Slice %)	58.9	72.2	95	0	0	1.8
Evaluator 2	Expert 2 (Slice %)	61	71.2	97	0	0	1.5
Evaluator 1	Expert 1 (Manual %)	60	70	92	0	0	0
Evaluator 2	Expert 2 (Manual %)	58	65	92	0	0	0
Evaluator 1	Non-expert 1 (Slice %)	86	93	96	11	3	31
Evaluator 2	Non-expert 2 (Slice %)	91	98	99	0	0	22
Evaluator 1	Non-expert 1 (Manual %)	10	30	80	0	0	1
Evaluator 2	Non-expert 2 (Manual %)	15	25	82	0	0	2

Supplementary Table 4: Validation 2: Absolute error (AE) calculation. ResNet CNN represents true values. AE Slice represents estimated values for Slice (%) classification. AE manual represents estimated values for manual rating (%). Evaluators were two experts and two non-experts.

Genotypes	ResNet CNN	AE Slice	AE Manual	Evaluators
V. thunbergii x *V. vinifera*	60	-1.1	0	Expert 1
Tigvoasa	68.4	4.1	1.6	Expert 1
Pinot Meunier	89	6	3	Expert 1
Riesling	0	0	0	Expert 1
Regent	0.4	-0.4	-0.4	Expert 1
Cabernet Sauvignon	2.2	-0.4	-2.2	Expert 1
V. thunbergii x *V. vinifera*	60	1	-2	Expert 2
Tigvoasa	68.4	2.8	-3.4	Expert 2
Pinot Meunier	89	8	3	Expert 2
Riesling	0	0	0	Expert 2
Regent	0.4	-0.4	-0.4	Expert 2
Cabernet Sauvignon	2.2	-0.7	-2.2	Expert 2
V. thunbergii x *V. vinifera*	60	26.7	-50	Non-expert 1
Tigvoasa	68.4	24.5	-38.4	Non-expert 1
Pinot Meunier	89	7.4	-9	Non-expert 1
Riesling	0	11	0	Non-expert 1
Regent	0.4	2.6	-0.4	Non-expert 1
Cabernet Sauvignon	2.2	29	-1.2	Non-expert 1
V. thunbergii x *V. vinifera*	60	31.5	-45	Non-expert 2
Tigvoasa	68.4	29.3	-43.4	Non-expert 2
Pinot Meunier	89	9.5	-7	Non-expert 2
Riesling	0	0	0	Non-expert 2
Regent	0.4	-0.4	-0.4	Non-expert 2
Cabernet Sauvignon	2.2	19.8	-0.2	Non-expert 2

Supplementary Table 5: Validation 3: Summary of audience manual rating of six genotypes. Genotypes with non-hairy leaves: ‘Riesling’, ‘Cabernet Sauvignon’ and ‘Regent’. Genotypes with hairy leaves: ‘Tigvoasa’, ‘Pinot Meunier’ and *V. thunbergii x V. vinifera*.

	**Riesling**	**Pinot Meunier**	**Cabernet Sauvignon**	**Tigvoasa**	**Regent**	***V. thunbergii* x *V. vinifera***
ResNet CNN %	0	89	2.2	68.4	0.4	60
Evaluator 1	0	80	2	15	0	20
Evaluator 2	0	100	10	80	0	50
Evaluator 3	1	70	0	20	0	10
Evaluator 4	0	70	5	25	0	15
Evaluator 5	20	80	30	50	15	70
Evaluator 6	0	100	0	30	0	10
Evaluator 7	20	100	20	0	20	50
Evaluator 8	0	96	0	63	0	52
Evaluator 9	0	75	0	5	0	2
Evaluator 10	0	80	7	20	5	27
Evaluator 11	0	80	10	20	0	40
Evaluator 12	0	90	5	40	0	20
Evaluator 13	10	90	25	50	20	35
Evaluator 14	0	80	0	40	20	30
Evaluator 15	0.5	98	5	25	3	30
Evaluator 16	0	98	0	50	0	30

Supplementary Table 6: Validation 3: Absolute error estimation of audience validation.

	**Riesling**	**Pinot Meunier**	**Cabernet Sauvignon**	**Tigvoasa**	**Regent**	***V. thunbergii* x *V. vinifera***
Evaluator 1	0	-19	-0.2	-68.4	-0.4	-8
Evaluator 2	0	-19	-0.2	-63.4	-0.4	-10
Evaluator 3	0	-14	-0.2	-53.4	-0.4	-10
Evaluator 4	0	-9	-0.2	-48.4	-0.4	-20
Evaluator 5	0	-9	-0.2	-48.4	-0.4	-25
Evaluator 6	0	-9	-0.2	-48.4	-0.4	-30
Evaluator 7	0	-9	1.8	-43.4	-0.4	-30
Evaluator 8	0	-9	4.8	-43.4	-0.4	-30
Evaluator 9	0	1	4.8	-38.4	-0.4	-33
Evaluator 10	0	1	4.8	-28.4	-0.4	-40
Evaluator 11	0	7	6.8	-28.4	2.6	-40
Evaluator 12	0	9	9.8	-18.4	4.6	-45
Evaluator 13	1	9	9.8	-18.4	14.6	-50
Evaluator 14	10	11	19.8	-18.4	19.6	-50
Evaluator 15	20	11	24.8	-5.4	19.6	-58
Evaluator 16	20	11	29.8	11.6	19.6	10
